# Carnosic Acid, a Natural Diterpene, Attenuates Arsenic-Induced Hepatotoxicity via Reducing Oxidative Stress, MAPK Activation, and Apoptotic Cell Death Pathway

**DOI:** 10.1155/2018/1421438

**Published:** 2018-05-02

**Authors:** Sonjit Das, Swarnalata Joardar, Prasenjit Manna, Tarun K. Dua, Niloy Bhattacharjee, Ritu Khanra, Shovonlal Bhowmick, Jatin Kalita, Achintya Saha, Supratim Ray, Vincenzo De Feo, Saikat Dewanjee

**Affiliations:** ^1^Advanced Pharmacognosy Research Laboratory, Department of Pharmaceutical Technology, Jadavpur University, Kolkata 700032, India; ^2^Biological Science and Technology Division, CSIR-North East Institute of Science and Technology, Jorhat, Assam 785006, India; ^3^Department of Chemical Technology, University of Calcutta, Kolkata 700009, India; ^4^Department of Pharmaceutical Sciences, Assam University, Silchar 788011, India; ^5^Department of Pharmacy, University of Salerno, Fisciano 84084, Italy

## Abstract

The present studies have been executed to explore the protective mechanism of carnosic acid (CA) against NaAsO_2_-induced hepatic injury. CA exhibited a concentration dependent (1–4 *μ*M) increase in cell viability against NaAsO_2_ (12 *μ*M) in murine hepatocytes. NaAsO_2_ treatment significantly enhanced the ROS-mediated oxidative stress in the hepatic cells both in *in vitro* and *in vivo* systems. Significant activation of MAPK, NF-*κ*B, p53, and intrinsic and extrinsic apoptotic signaling was observed in NaAsO_2_-exposed hepatic cells. CA could significantly counteract with redox stress and ROS-mediated signaling and thereby attenuated NaAsO_2_-mediated hepatotoxicity. NaAsO_2_ (10 mg/kg) treatment caused significant increment in the As bioaccumulation, cytosolic ATP level, DNA fragmentation, and oxidation in the liver of experimental mice (*n* = 6). The serum biochemical and haematological parameters were significantly altered in the NaAsO_2_-exposed mice (*n* = 6). Simultaneous treatment with CA (10 and 20 mg/kg) could significantly reinstate the NaAsO_2_-mediated toxicological effects in the liver. Molecular docking and dynamics predicted the possible interaction patterns and the stability of interactions between CA and signal proteins. ADME prediction anticipated the drug-likeness characteristics of CA. Hence, there would be an option to employ CA as a new therapeutic agent against As-mediated toxic manifestations in future.

## 1. Introduction

Arsenic (As) is a toxic metalloid, which raises much disquiet in the health standpoints for human and animals [1]. The primary natural pools of As are rocks, from which As is mobilized through natural different processes [2]. Besides the natural sources, industrial outcomes can also cause the release and mobilization of As to soil, water, and air in various forms [2, 3]. Organic As compounds are not the concern for health risks [4]. Inorganic trivalent arsenicals (arsenites, AsO_2_^−^) are most potent toxicants [5]. Drinking water contaminated with arsenites is thought to be the major root of As calamity affecting >140 million people in ∼70 countries [4]. Following ingestion, As is absorbed through the gastrointestinal tract and bioaccumulated into various organs [6]. It can also enter into the body through the respiratory system and dermis [7]. Intake of arsenites > 30 *μ*g per day has been reported to exert arsenicosis to the critical organs [4, 6, 8]. Earlier investigations revealed that As reduces mitochondrial integrity, resulting in random formation of superoxide radical which subsequently potentiates a cascade of radical reactions and enhances the secondary generation of other ROS [6, 7, 9, 10]. On the other hand, As further promotes oxidative stress by reacting with -SH group and thereby deactivates the defense mechanism of several antioxidant enzymes and glutathione system [5]. The excess of ROS triggers wide ranges of pathological occurrences including damages to the structural components of cells, alteration in the expressions of genes, DNA damage, and apoptosis [6, 7]. Despite As is a major threat to the global health, the development of a suitable therapy is still underway. The primary therapeutic strategy includes the treatment with chelating agents; however, the adverse effects like removal of essential metals and redistribution of As largely restricted their clinical usefulness [6]. The biological half-life of inorganic As is ∼10 h [11], which also argues against the employment of chelating agent as therapeutic negotiator. As-mediated augmented oxidative stress has been considered to be the principle etiology of arsenicosis. Therefore, it would be worthy to exploit the role of natural antioxidants to counteract As-mediated toxic manifestations.

**Figure 1:**
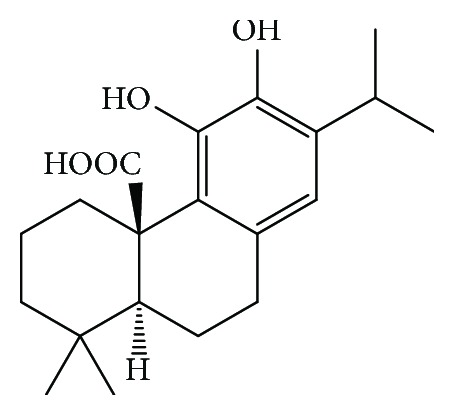
Structure of carnosic acid.

Carnosic acid (CA), (4aR,10aS)-5,6-dihydroxy-1,1-dimethyl-7-propan-2-yl-2,3,4,9,10,10a-hexahydrophenanthrene-4a-carboxylic acid, is a naturally occurring phenolic diterpene (Figure 1). CA is commonly found in *Rosmarinus officinalis* and *Salvia officinalis* [12, 13]. CA has been reported to possess antioxidant [14, 15], neuroprotective [16], antiobesity [17], and anti-inflammatory [18] activities. Considering the antioxidant and radical scavenging effects of CA, the present studies have been undertaken to evaluate the possible therapeutic role of CA against As-induced hepatotoxicity.

## 2. Material and Methods

### 2.1. Chemicals

CA, bovine serum albumin (BSA), Dulbecco's modified Eagle's medium (DMEM), fetal bovine serum (FBS), Bradford reagent, and collagenase type I were procured from Sigma-Aldrich Chemical Company, MO, USA. Antibodies for immunoblotting were bought from Novus Biologicals, CO, USA. The solvents (HPLC grade) were obtained from Merck, Mumbai, India. Kits for the measurement of different biochemical parameters were procured from Span Diagnostics Ltd., India. 1-Chloro-2,4-dinitrobenzene, (NH_4_)_2_SO_4_, NaAsO_2_, 2,4-dinitrophenylhydrazine, ethylenediaminetetraacetic acid, 5,5-dithiobis(2-nitrobenzoic acid), N-Ethylmaleimide, nitro blue tetrazolium, NADH, KH_2_PO_4_, phenazine methosulphate, Na_4_P_2_O_7_, GSH, NaN_3_, thiobarbituric acid, 5-thio-2-nitrobenzoic acid, and CCl_3_COOH were obtained from Sisco Research Laboratory, Mumbai, India.

### 2.2. Animals

Healthy Swiss albino mice (♂, ∼2 month old, 25 ± 5 g) were procured from Chakraborty Enterprise, Kolkata, India and were housed in standard polypropylene cages (29 × 22 × 14 cm) in the animal house of the Department of Pharmaceutical Technology, Jadavpur University, India. The mice were maintained with temperature (22 ± 2°C), humidity (40 ± 10%), and 12 h light–dark cycle [19]. The mice were fed standard diet and water ad libitum. The *in vivo* experiment has been permitted (Ref number AEC/PHARM/1701/09/2017) by the animal ethical committee, Jadavpur University (Reg. number: 0367/01/C/CPCSEA, UGC, India), and the principles of laboratory animal care were followed during the experiment [20]. The animals were acclimatized for a period of 2 weeks before the execution of the *in vivo* experiment.

### 2.3. *In Vitro* Assays

#### 2.3.1. Determination of Cytotoxic Effect of NaAsO_2_

Hepatocytes were isolated from the liver of immediately sacrificed albino mice by two-step in situ collagenase perfusion as described by Dua et al. [6]. The hepatocytes were passaged at least a couple of times before the execution of *in vitro* experiment. Concentration-dependent cytotoxic effect of NaAsO_2_ was determined. Briefly, hepatocytes (~2 × 10^6^ cells/well) were seeded in tissue culture plate and incubated at 37°C and 5% CO_2_ for 24 h to form uniform monolayer of hepatocytes in the wells of culture plate. The cells were exposed to different concentrations of NaAsO_2_ for 2 h, and the cell viability was determined employing MTT (3-(4,5-dimethylthiazolyl-2)-2, 5-diphenyltetrazolium bromide) assay [6]. The experiments were performed in triplicate. NaAsO_2_ exhibited IC_50_ value of ∼12 *μ*M in murine hepatocytes.

#### 2.3.2. Determination of Effect of CA on Murine Hepatocytes

To determine the effect of CA on hepatocytes, hepatocytes (~2 × 10^6^ cells/set) were exposed to CA (1, 2, 4, 6, and 10 *μ*M). The experiments were performed in triplicate. The cell viability was measured employing MTT assay at different intervals up to 4 h [6].

#### 2.3.3. Assessment of Cytoprotective Role of CA

To determine the cytoprotective effect of CA, hepatocytes (~2 × 10^6^ cells/set) were exposed to NaAsO_2_ (12 *μ*M) along with CA (1, 2, 4, 6 and 10 *μ*M). The experiments were performed in triplicate. The cell viability was measured employing MTT assay at different intervals up to 4 h [6]. One set of hepatocytes exposed to NaAsO_2_ (12 *μ*M) was kept as toxic control, while an untreated set of hepatocytes was maintained as untreated control.

#### 2.3.4. Hoechst Staining

Hoechst 33258 nuclear staining has been executed to study the cytotoxic events [21]. Briefly, hepatocytes (~2000 cells/well) were exposed to NaAsO_2_ (12 *μ*M) and NaAsO_2_ (12 *μ*M) along with CA (4 *μ*M) for 2 h at 37°C and 5% CO_2_. One set of hepatocytes without any treatment was kept as normal control. After 4 h, hepatocytes were fixed with paraformaldehyde (4%) in phosphate buffer saline (PBS) of pH 7.4 for 20 min. The fixed hepatocytes were exposed to Hoechst 33258 (5 *μ*g/ml in PBS) for 15 min followed by washing with PBS. Fluorescent nuclei and nuclear pattern were recorded. The experiments were performed in triplicate.

#### 2.3.5. Flow Cytometric Analysis

The flow cytometric study has been performed to accomplish the nature of cell death. Briefly, hepatocytes were exposed to NaAsO_2_ (12 *μ*M) and NaAsO_2_ (12 *μ*M) along with CA (4 *μ*M) for 2 h at 37°C and 5% CO_2_. One set of hepatocytes without any treatment was kept as normal control. After 2 h, different sets of hepatocytes were treated with propidium iodide (PI) and FITC-labeled annexin V for 30 min at 37°C [21]. The excess of PI and annexin V was washed out, and the cells were fixed for analyzing in a flow cytometer using FACSCalibur (Becton Dickinson, Mountain View, USA) equipped with 488 nm argon laser light source; 515 nm band pass filter for FITC fluorescence and 623 nm band pass filter for PI fluorescence using CellQuest software, USA. The scatter plots of PI fluorescence (*y*-axis) versus FITC fluorescence (*x*-axis) were prepared for different sets of hepatocytes. The experiments were performed in triplicate.

#### 2.3.6. Assays for Redox Markers

Different sets of hepatocytes, each containing 1 ml of suspension (~2 × 10^6^ cells/ml), were used in experiments. The prophylactic role of CA against NaAsO_2_ intoxication was analyzed by incubating hepatocytes with CA (4 *μ*M) and NaAsO_2_ (12 *μ*M) together for 2 h at 37°C. One set of hepatocytes incubated with NaAsO_2_ (12 *μ*M) served as toxic control. One set of hepatocytes without any treatment was kept as normal control. The intracellular ROS production was estimated by measuring the fluorescence of 2,7-dichlorofluorescein diacetate (DCF) in a fluorescence spectrometer (Olympus 1X70, Japan) following the method as described by Manna and Jain [22]. The levels of lipid peroxidation, protein carbonylation, reduced glutathione (GSH), and the levels of endogenous antioxidant enzymes, namely superoxide dismutase (SOD), catalase (CAT), glutathione peroxidase (GPx), glutathione-S-transferase (GST), and glutathione reductase (GR), were quantified employing reputable protocols [23]. The experiments were performed in triplicate.

#### 2.3.7. Western Blotting of Signal Proteins in the Hepatocytes

The protein samples of hepatocytes for specific cellular components namely whole cell, cytosolic, and mitochondrial fractions were separated following standard sequential fractionation procedure as described by Baghirova and coworkers [24]. The sample proteins (20 *μ*g) were resolved in 12% SDS-PAGE gel electrophoresis and transferred into nitrocellulose membrane [25, 26]. The membrane was blocked by blocking buffer (containing 5% nonfat dry milk) for 1 h and subsequently incubated with primary antibody at 4°C overnight. After primary antibody treatment, the membrane was washed with tris-buffered saline (TBST; containing 0.1% tween 20). The membrane was then treated with suitable HRP-conjugated secondary antibody at room temperature for 1 h. The blots were developed by ECL substrate (Millipore, MA, USA) to detect the expressions of proteins in a ChemiDoc Touch Imaging System (Bio-Rad, USA). The densitometric analysis was performed using Image Lab software (Bio-Rad, USA). Normalization of expression was done employing *β*-actin as a loading protein. The membranes were then subjected to mild stripping using stripping buffer containing 1% SDS (pH 2.0) and glycine (25 mM), followed by treatments with respective primary and secondary antibodies for detecting the expressions of other proteins in the same membrane. The expressions of Bcl-2, Bax, cytochrome C, Apaf-1, cleaved caspase 9, cleaved caspase 3, Bid, Fas, cleaved caspase 8, total JNK, phospho-JNK (Tyr 183/Tyr 185), total-p38, phospho-p38 (Tyr 180/Tyr 182), p53, phospho-I*κ*B*α* (Ser 32), and total I*κ*Bα, phospho-NF-*κ*B (p65) (Ser 536) were estimated. The experiments were performed in triplicate.

### 2.4. *In Vivo* Bioassay

#### 2.4.1. Experimental Setup

The *in vivo* experiment was performed following established protocol by our group [6.7]. Twenty-four Swiss albino mice (♂) were divided into four groups (*n* = 6) and were treated as follows:

Gr I: normal control, mice received only 1% tween 80 in distilled water (1 ml, *p.o.*, once daily) for 15 days.

Gr II: toxic control, mice were treated with NaAsO_2_ (10 mg/kg body weight, *p.o.*, once daily) for 10 days.

Gr III: animals were treated with CA (10 mg/kg body weight, *p.o.*, once daily for 15 days) began 5 days prior to the beginning of exposure to NaAsO_2_ (10 mg/kg body weight, *p.o.*, once daily for 10 days), totalizing 15 days treatment period.

Gr IV: animals were treated with CA (20 mg/kg body weight, *p.o.*, once daily for 15 days) began 5 days prior to the beginning of exposure to NaAsO_2_ (10 mg/kg body weight, *p.o.*, once daily for 10 days), totalizing 15 days treatment period.

The doses of CA were selected on the basis of *in vitro* observation, subacute toxicity studies, preliminary *in vivo* study (with limited number of animals). In subacute toxicity studies, CA (10 and 20 mg/kg body weight, *p.o.*, once daily for 30 days) did not show any significant change in haematological, biochemical, and histological parameters when compared with normal mice (Supplementary Table 2 and Supplementary Figure 2).

**Figure 2:**
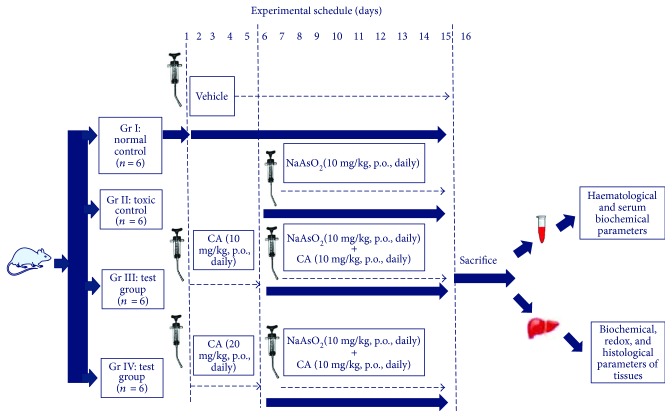
A schematic outline of *in vivo* experimental protocol.

The food intake and water intake were monitored on a daily basis. After 15 days, the mice were fasted overnight and were sacrificed by cervical dislocation under CO_2_ anesthesia. Before sacrificing the mice, body weight was recorded. For measurements of haematological parameters and biochemical markers in the sera, blood samples were collected from retro-orbital venous plexus after applying tetracaine (0.5%, one drop) ophthalmic anesthetic drop to the eyes. The livers were excised and cleaned with PBS. The weight of liver was recorded. The organs were homogenized immediately in Tris–HCl (0.01 M) + EDTA (0.001 M) buffers of pH 7.4 and centrifuged (12,000*g*) at 4°C for 30 min to obtain tissue homogenate. Urine samples were collected from the bladder and immediately stored at −80°C [27]. A schematic overview of the *in vivo* assay has been depicted in Figure 2.

#### 2.4.2. Estimation of Haematological and Serum Biochemical Parameters

Total erythrocyte count was measured using a haemocytometer and haemoglobin content was estimated using a haemoglobinometer. The levels of alanine aminotransferase (ALT), aspartate aminotransferase (AST), creatine kinase (CK), and lactate dehydrogenase (LDH) in the sera were estimated by commercially available kits (Span Diagnostics Limited, India) following manufacturer's protocol.

#### 2.4.3. Assays for Hepatic and Urinary As

The As contents in liver and urine of experimental animals were analyzed following the method of Das et al. [28], using hydride generation system in atomic absorption spectrophotometer (Perkin Elmer model number 3100, USA).

#### 2.4.4. Assays for Biochemical and Redox Markers in Liver

Cytosolic fractions from livers of experimental mice were isolated following subcellular fractionation protocol employing centrifugation methods mentioned by Abcam, Cambridge, MA, USA. Cytosolic ATP level in the hepatic tissue was estimated by the commercially available assay kit (Abcam, Cambridge, MA, USA) following manufacturer's protocol. The extent of DNA fragmentation was measured by the diphenylamine reaction as described by Lin et al. [29]. Briefly, the hepatic tissue was lysed with hypotonic lysing buffer (10 mM Tris, pH 8.0, 1 mM EDTA, 0.5% triton X-100), and the lysates were centrifuged to separate intact and fragmented fractions. Then the pellet and the supernatant were separately precipitated with 12.5% trichloroacetic acid. The DNA precipitates were heated to 90°C for 10 min in 5% trichloroacetic acid and quantitatively estimated by colorimetric reaction with diphenylamine [29]. DNA oxidation was evaluated by RP-HPLC analysis and was represented as 7,8-hydroxy-2′-deoxyguaosine/2′-deoxyguaosine (8-OHdG/2-dG) ratio [30, 31]. Briefly, DNA was isolated from hepatic tissue by pronase-ethanol method followed by enzymatic digestion as the protocol by Chepelev et al. [31]. 8-OHdG stock solution (35 mmol/l) was prepared in water, while 2-dG stock solution (37 mmol/l) was prepared in 0.5 mol/l NH_4_OH [30]. The aliquots of 8-OHdG and 2-dG stock solutions were adequately diluted first in water and finally in digested DNA samples to obtain working spiked solutions. Quantitative estimation was done in Dionex UltiMate 3000 HPLC system (Dionex, Germany), using a C-18 column (250 × 4.6 mm, particle size 5 *μ*) and electrochemical detector. The columns were preconditioned with 1 ml acetonitrile, followed by 1 ml water, and 1 ml 100 mM NaH_2_PO_4_ (pH 6.0). For 8-OHdG analysis, the mobile phase (pH 3.0) contained formic acid (0.1 mol/l), citric acid (1.0 mmol/l), NaN_3_ (7.7 mmol/l), EDTA (0.5 mmol/l), diethylamine (24.0 mmol/l), and 4% acetonitrile. For 2-dG analysis, the mobile phase comprised NaH_2_PO_4_ (50.0 mmol/l) (pH 4.5) and 4% acetonitrile. A flow rate of 1.0 ml/min was used. The applied potentials at the first and second electrodes for 8-OHdG were 0.0 and+ 0.5 volts, respectively. While the applied potentials for 2-dG were +0.4 and +0.8 volts at the first and second electrodes, respectively. The intercellular ROS, TBARS level, protein carbonylation, endogenous antioxidant enzymes, and GSH levels were assayed following established protocols [23].

#### 2.4.5. Western Blotting of Signaling Proteins in Liver

The protein samples of liver for specific cellular components, namely cytosolic, mitochondrial, and nuclear fractions, were separated, employing sequential fractionation process [24]. The sample proteins (20 *μ*g) were resolved in 12% SDS-PAGE gel electrophoresis and immunoblotted as mentioned in the earlier section. The expressions of Bcl-2, Bax, cytochrome C, Apaf-1, cleaved caspase 9, cleaved caspase 3, Bid, Fas, cleaved caspase 8, total JNK, phospho-JNK (Tyr 183/Tyr 185), total-p38, phospho-p38 (Tyr 180/Tyr 182), p53, phospho-I*κ*B*α* (Ser 32), total I*κ*Bα, and phospho-NF-*κ*B (p65) (Ser 536) were estimated.

#### 2.4.6. Histological Studies

The livers from the experimental mice were immediately fixed in 10% buffered formalin and were processed for paraffin sectioning. Sections (~5 *μ*m) were stained with hematoxylin and eosin (H & E) for studying the histology of hepatic tissues [32].

#### 2.4.7. Statistical Analysis

The experimental data were denoted as mean ± SD. The data were statistically examined by one-way ANOVA followed by Dunnett's *t*-test using computerized GraphPad InStat (version 3.05), GraphPad software, USA. The values were considered significant when *p* < 0.05 or 0.01.

### 2.5. In Silico Prediction

#### 2.5.1. Absorption, Distribution, Metabolism, and Excretion (ADME) Prediction

In silico ADME properties of CA were predicted using QikProp module of Maestro Schrödinger software [33]. Lipinski's rule of five (molecular weight < 500, number of hydrogen bond donors < 5, number of hydrogen bond acceptors < 10, log*P* < 5) was also assessed, which helped in distinguishing between drug-like or non-drug-like profiles of a candidate molecule. Furthermore, some important ADME profiles, like oral absorptions, cell permeability, blood brain barrier, and so on of CA, were predicted in silico.

#### 2.5.2. Molecular Docking and Postdocking Simulation Analysis

The high-resolution X-ray crystallographic structures of proteins were retrieved from Protein Data Bank (PDB) accessed on June 2017 [34]. The protein crystal structures for Bax (PDB ID: 4BD2), Bcl-2 (PDB: 4LXD), cytochrome C (PDB: 3ZCF), Apaf-1 (PDB: 1Z6T), caspase 9 (PDB: 2AR9), caspase 3 (PDB: 5I9B), Fas (PDB: 3EZQ), caspase 8 (PDB: 4PRZ), Bid (PDB: 4QVE), I*κ*B (PDB: 4KIK), JNK (PDB: 4E73), NF-*κ*B (PDB: 4IDV), p53 (PDB: 2XWR), and p38 (PDB: 3S3I) were downloaded in PDB format. To perform molecular docking, all proteins were prepared in Protein Preparation Wizard using Maestro in the Schrödinger suite [33] for addition of hydrogen atoms and to repair the faults like missing loops and steric clashes. Hence, protonation state was selected to be consistent in physiological pH. To obtain the minimum energy structure, system was subjected to a restrained minimization using the OPLS2005 force field in Impact Refinement module (Impref). The ligand, CA, was processed in LigPrep module of Schrödinger [33]. Using the Receptor Grid generation panel, the receptor grid was generated. During the grid generation step, the binding site was defined by a rectangular box surrounding the cocrystallized ligand. The position and size of the active site were also determined. Molecular docking was performed using standard precision (SP) method of docking using Glide module [33] to evaluate the binding affinity between protein and ligand.

Following molecular docking, the protein-ligand complex was subjected to molecular dynamics (MD) simulation study using the Desmond program (Desmond Molecular Dynamics System and Maestro-Desmond Interoperability Tools, Schrödinger) [33]. The complex was neutralized by appropriate number of ions and surrounded by an orthorhombic shape of water box solvated by TIP3P (transferable intermolecular potential 3P) water model [35]. By adopting the SHAKE algorithm, bond lengths to H atoms and internal geometry of H_2_O molecules were constrained [36]. The simulation was carried out for each complex in equilibrated system of NPT ensemble at maximum 40 ns using the Nose-Hoover chain thermostat temperature at 300 K and Martyna-Tobias-Klein barostat bar pressure at 1.013 with a relaxation time of 1.0 ps and 2.0 ps, respectively. Energy of system and atomic coordinate data were recorded in every 1.2 ps time intervals.

## 3. Results

### 3.1. Effect of CA against NaAsO_2_ Intoxication *In Vitro*

#### 3.1.1. Dose-Dependent Effect of NaAsO_2_-Induced Cytotoxicity

**Figure 3:**
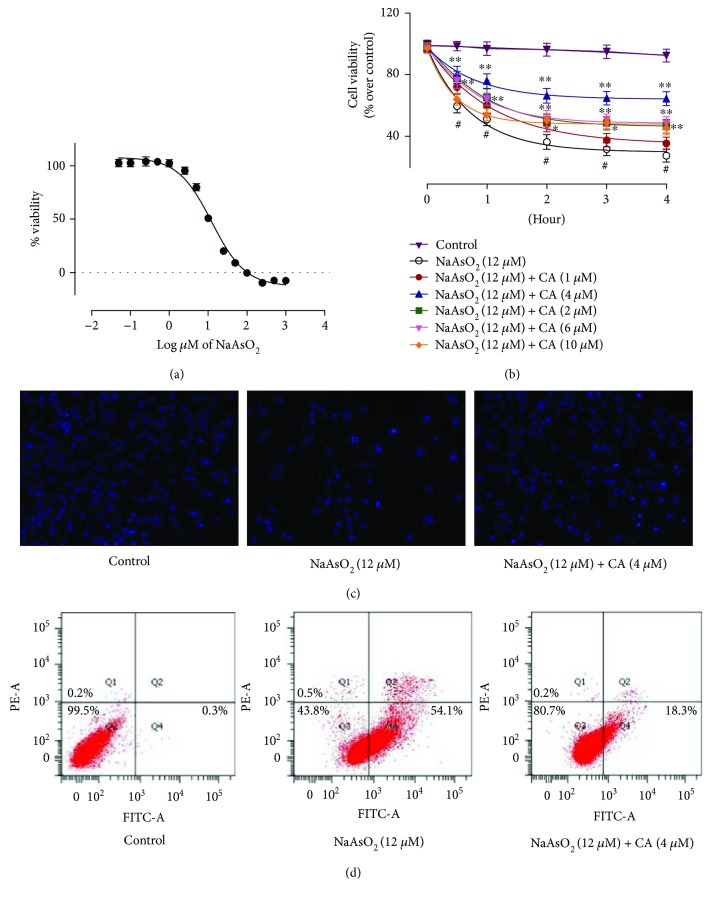
The cell viability, image, and flow cytometric assays in the absence (NaAsO_2_) and presence of CA (NaAsO_2_ + CA) *in vitro* employing isolated murine hepatocytes. (a) Effect of NaAsO_2_ at different concentrations on cell viability in isolated murine hepatocytes. Values are represented as mean ± SD (*n* = 3). (b) Effect on cell viability in the absence (NaAsO_2_) and presence of CA (NaAsO_2_ + CA). Values are represented as mean ± SD (*n* = 3). ^#^Values significantly (*p* < 0.01) differ from normal control. ^∗^Values significantly (*p* < 0.05) differ from toxic control. ^∗∗^Values significantly (*p* < 0.01) differ from toxic control. (c) Hoechst staining of hepatocytes in the absence (NaAsO_2_) and presence of CA (NaAsO_2_ + CA). (d) Percentage distribution of apoptotic and necrotic cells in the absence (NaAsO_2_) and presence of CA (NaAsO_2_ + CA) analyzed by flow cytometric assay.

Reduction of cell viability is the index of cytotoxicity. In search of the cytotoxic effect of NaAsO_2_, hepatocytes were incubated with NaAsO_2_ at different concentrations for 2 h. The cell viability was reduced by NaAsO_2_ in a concentration-dependent manner (Figure 3(a)). The IC_50_ value has been found to be 11.6 *μ*M (~12 *μ*M). Based on the observed IC_50_ value, subsequent *in vitro* experiments were executed using NaAsO_2_ (12 *μ*M) as a toxic control.

#### 3.1.2. Effect of CA on Murine Hepatocytes

The hepatocytes incubated with CA (1–6 *μ*M) did not show any significant change in the cell viability up to 4 h of experimental duration when compared with normal/untreated hepatocytes (∼95.2% at 4 h). However, cell viability was slightly/insignificantly reduced (∼89.2% at 4 h) with CA (10 *μ*M). The data were included as supplementary Figure 1.

#### 3.1.3. Concentration-Dependent Cytoprotective Role of CA against NaAsO_2_-Induced Cytotoxicity

NaAsO_2_ (12 *μ*M, ~IC_50_) exposed hepatocytes exhibited significant (*p* < 0.01) reduction in cell viability up to 4 h (Figure 3(b)). Simultaneous treatment of hepatocytes with CA (1–10 *μ*M) and NaAsO_2_ (12 *μ*M) significantly (*p* < 0.05–0.01) prevented the reduction in cell viability in a concentration-dependent manner up to 4 *μ*M. However, cytoprotective effect of CA was further reduced >4 *μ*M concentration when compared with CA (4 *μ*M). Based on the observation, the concentration of CA and the incubation time have been standardized to 4 *μ*M and 2 h, respectively, for subsequent *in vitro* experiments. The CA (4 *μ*M) alone did not show any significant difference in redox parameters in isolated murine hepatocytes (Supplementary Table 1).

#### 3.1.4. Effect on Hoechst Staining

The cytoprotective effect of CA has been estimated by Hoechst staining following visualization through fluorescence microscope (Figure 3(c)) of hepatocytes under different treatments. NaAsO_2_ (12 *μ*M) treated hepatocytes exhibited significant reduction of visible nuclei; however, the visible nuclei exhibited explicit patterns of morphological changes, condensation, fragmentation of the nuclei, and chromatin condensation. Simultaneous incubation of hepatocytes with CA (4 *μ*M) and NaAsO_2_ (12 *μ*M) could significantly attenuate the cytotoxic effect of NaAsO_2_ (12 *μ*M) and could restore nuclear morphology to near-normal status.

#### 3.1.5. Flow Cytometric Analysis

To investigate the nature of cell death, hepatocytes under different treatments were assessed by flow cytometric analysis. Flow cytometric data (Figure 3(d)) revealed that NaAsO_2_ (12 *μ*M) treated hepatocytes exhibited very low PI staining (~0.5%) with very high annexin V-FITC binding (~54.1%) indicating majority of apoptotic cells. Simultaneous treatment of hepatocytes with CA (4 *μ*M) and NaAsO_2_ (12 *μ*M) resulted significant reduction in the count of the apoptotic cells (~18.3%) indicating a possible cytoprotective effect of CA against NaAsO_2_ (12 *μ*M) induced cytotoxicity. The control group showed very little apoptotic (~0.3%) and necrotic (~0.2%) cells as compared with viable cells (~99.5%).

#### 3.1.6. Effects on NaAsO_2_-Induced Alteration in Redox Status in Hepatocytes

**Figure 4:**
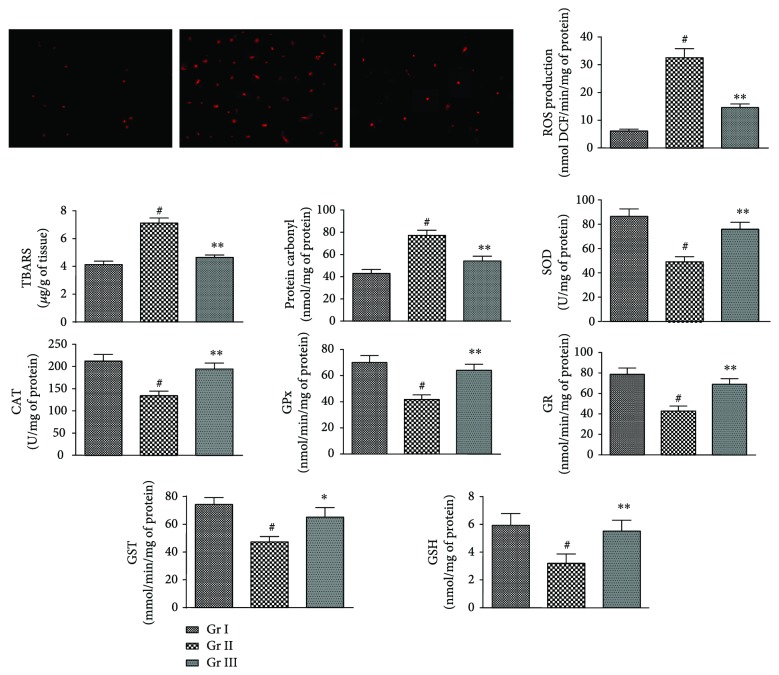
The effect on ROS accumulation, lipid peroxidation, protein carbonylation, and endogenous redox systems in the absence (NaAsO_2_) and presence of CA (NaAsO_2_ + CA) in isolated murine hepatocytes. Values are represented as mean ± SD (*n* = 3). ^#^Values significantly (*p* < 0.01) differ from normal control. ^∗^Values significantly (*p* < 0.05) differ from toxic control. ^∗∗^Values significantly (*p* < 0.01) differ from toxic control. SOD unit, “U” is defined as inhibition (*μ* moles) of NBT-reduction/min. CAT unit “U” is defined as H_2_O_2_ consumption/min. Gr I: normal control; Gr II: toxic control; Gr III: hepatocytes incubated with NaAsO_2_ (12 *μ*M) along with CA (4 *μ*M).

The prophylactic effects of CA on NaAsO_2_-induced oxidative stress in isolated murine hepatocyte have been depicted in Figure 4. Overproduction of ROS is an index of redox-challenged cellular atmosphere. In this study, NaAsO_2_ (12 *μ*M) incubated hepatocytes revealed significant (*p* < 0.01) enhance in ROS production in the hepatocytes as ostensible from fluorescence of DCF measured in fluorescence microscope. CA (4 *μ*M) could significantly (*p* < 0.01) reduce the NaAsO_2_ (12 *μ*M) mediated ROS production in the hepatocytes. Overproduction of ROS causes oxidative damage of cellular macromolecules. In this study, NaAsO_2_ (12 *μ*M) exposed hepatocytes exhibited significantly (*p* < 0.01) high level of thiobarbituric acid reactive substances (TBARS) level. TBARS, a by-product of lipid peroxidation, is used as a marker to estimate the extent of lipid peroxidation. NaAsO_2_ (12 *μ*M) exposed hepatocytes showed significant (*p* < 0.01) escalation in the level of carbonylated protein. However, simultaneous treatment with CA (4 *μ*M) could significantly attenuate NaAsO_2_ mediated the lipid peroxidation (<0.01) and protein carbonylation (<0.01). NaAsO_2_ (12 *μ*M) intoxication concomitantly creates a redox-challenged cellular environment via significant (<0.01) depletion in the levels of endogenous antioxidant enzymes (SOD, CAT, GPx, GST, and GR) and GSH. However, treatment with CA (4 *μ*M) along with NaAsO_2_ (12 *μ*M) could significantly reverse the NaAsO_2_-mediated changes in the levels of antioxidant enzymes (*p* < 0.05–0.01) and GSH (*p* < 0.01) in the murine hepatocytes.

#### 3.1.7. Effects on Apoptotic Events in Hepatocytes

**Figure 5:**
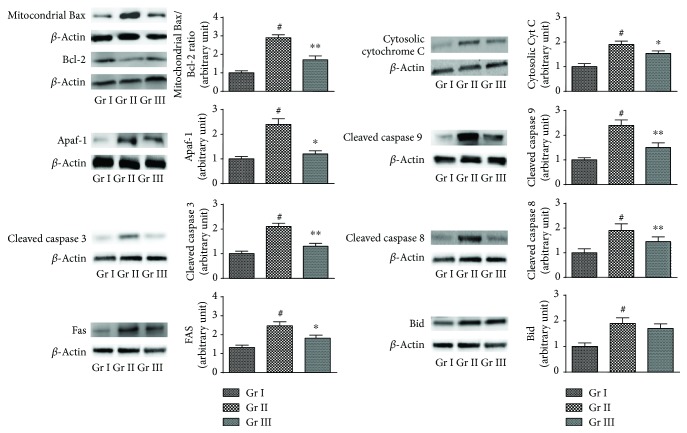
The effect on intrinsic and extrinsic apoptotic signaling in the absence (NaAsO_2_) and presence of CA (NaAsO_2_ + CA) *in vitro*. The relative band intensities were assessed and the normal control band was allotted a random value of 1. *β*-actin served as loading control. Values are expressed as mean ± SD (*n* = 3). ^#^Values significantly (*p* < 0.01) differ from normal control. ^∗^Values significantly (*p* < 0.05) differ from toxic control. ^∗∗^Values significantly (*p* < 0.01) differ from toxic control. Gr I: normal control; Gr II: toxic control; Gr III: hepatocytes incubated with NaAsO_2_ (12 *μ*M) along with CA (4 *μ*M).

The prophylactic effects of CA on NaAsO_2_-induced intrinsic and extrinsic apoptotic signaling have been shown in Figure 5. Oxidative stress further promotes apoptosis via downregulation and upregulation in the transcriptions of antiapoptotic and proapoptotic signal proteins, respectively. In this study, the Western blot analysis revealed that NaAsO_2_ (12 *μ*M) caused significant upregulation in the expression of the Bax (proapoptotic) protein in mitochondria with concomitant downregulation in the expression of Bcl-2 (antiapoptotic) protein, resulting a significant (*p* < 0.01) increase in Bax/Bcl-2 ratio in the hepatocytes. NaAsO_2_ (12 *μ*M) exposure further promoted the release of cytochrome C to the cytosol evidenced from significant upregulation (*p* < 0.01) in the expression of cytosolic cytochrome C in murine hepatocytes. NaAsO_2_ (12 *μ*M) treatment significantly (*p* < 0.01) activated Apaf-1 expression in the cytosol of murine hepatocytes, which further endorsed the cleavage of procaspases. Significant upregulations (*p* < 0.01) in the expressions of cleaved caspase 9 and cleaved caspase 3 were observed in NaAsO_2_ (12 *μ*M) exposed hepatocytes. However, CA (4 *μ*M) cotreatment could significantly (*p* < 0.05–0.01) reinstate the expressions of aforementioned intrinsic apoptotic proteins to near-normal status. In search of the effect on extrinsic pathway of apoptosis (mitochondria independent), Western blot analysis of FAS, cleaved caspase 8, and Bid were performed. NaAsO_2_ (12 *μ*M) exposed hepatocytes exhibited significant (*p* < 0.01) upregulations in the expressions of FAS, cleaved caspase 8, and Bid. CA (4 *μ*M) cotreatment could significantly restore the expressions of FAS (*p* < 0.05) and cleaved caspase 8 (*p* < 0.01) to near-normal status. However, CA (4 *μ*M) was not found to be effective against Bid.

**Figure 6:**
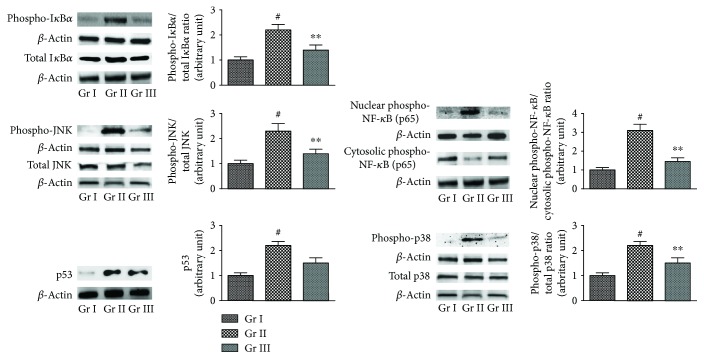
The effect on I*κ*B*α*, NF-*κ*B, JNK, p38, and p53 signaling in the absence (NaAsO_2_) and presence of CA (NaAsO_2_ + CA) *in vitro*. The relative band intensities were assessed and the normal control band was allotted a random value of 1. *β*-actin served as loading control. Values are expressed as mean ± SD (*n* = 3). ^#^Values significantly (*p* < 0.01) differ from normal control. ^∗^Values significantly (*p* < 0.05) differ from toxic control. ^∗∗^Values significantly (*p* < 0.01) differ from toxic control. Gr I: normal control; Gr II: toxic control; Gr III: hepatocytes incubated with NaAsO_2_ (12 *μ*M) along with CA (4 *μ*M).

To investigate the proapoptotic effect of NF-*κ*B, the signal proteins were immunoblotted (Figure 6). NaAsO_2_ (12 *μ*M) treatment could significantly (*p* < 0.01) stimulate the phosphorylation of cytosolic I*κ*B*κ* and thereby could activate NF-*κ*B (p65) signaling. A significant (*p* < 0.01) translocation of NF-*κ*B (p65) to the nucleus was visualized in NaAsO_2_ (12 *μ*M) exposed hepatocytes. However, CA (4 *μ*M) could significantly (*p* < 0.01) attenuate the aforementioned NF-*κ*B signaling pathway within the hepatocytes. ROS can also trigger the mitogen-activated protein kinases (MAPK). In this study, NaAsO_2_ (12 *μ*M) could significantly (*p* < 0.01) stimulate the phosphorylation of JNK and p38 (Figure 6). However, CA (4 *μ*M) could significantly (*p* < 0.01) downregulate the NaAsO_2_ (12 *μ*M) mediated phosphorylation of JNK and p38. On the other hand, NaAsO_2_ (12 *μ*M) could significantly (*p* < 0.01) upregulate p53 signaling, while CA (4 *μ*M) was not found to be effective against p53.

### 3.2. Effects of CA against NaAsO_2_ Intoxication *In Vivo*

#### 3.2.1. Effect on Body Weight, Liver Weight, Hepatic As Content, and Urinary As Content

During the tenure of experiment, no significant change in food and water intake was recorded in the animals of either of experimental group. NaAsO_2_ (10 mg/kg) treated mice exhibited significant (*p* < 0.01) reduction in the body weight; however, simultaneous administration of CA (10 and 20 mg/kg) along with NaAsO_2_ (10 mg/kg) could significantly (*p* < 0.05) restore the body weight to near-normal status (Table 1). No significant difference in liver weight was observed in the experimental mice of either group. NaAsO_2_ (10 mg/kg) exposed mice exhibited significant (*p* < 0.01) increase in the accumulation of As in the liver coupled with significantly (*p* < 0.01) poor urinary clearance of As when compared with normal mice. On the other hand, simultaneous administration of CA (10 and 20 mg/kg) along with NaAsO_2_ (10 mg/kg) could significantly (*p* < 0.05–0.01) promote the As clearance resulting significantly (*p* < 0.05) low hepatic As content.

#### 3.2.2. Effects on Blood Parameters

Blood parameters give crucial impression of pathological state within the system. The effects of CA on blood parameters of experimental mice were reported in Table 2. NaAsO_2_ (10 mg/kg) treated mice exhibited significant (*p* < 0.01) reduction in erythrocyte counts and haemoglobin level. However, simultaneous administration of CA (10 and 20 mg/kg) along with NaAsO_2_ (10 mg/kg) could significantly (*p* < 0.05–0.01) restore the erythrocyte counts and haemoglobin level (*p* < 0.01) to near-normal status. NaAsO_2_ (10 mg/kg) treatment caused significant (*p* < 0.01) increase in ALT, AST, LDH, and CK levels in the sera of experimental mice. However, concurrent administration of CA (10 and 20 mg/kg) along with NaAsO_2_ (10 mg/kg) could significantly (*p* < 0.05–0.01) revert the serum biochemical parameters to near-normal status.

#### 3.2.3. Effects on Redox Status in Liver

**Figure 7:**
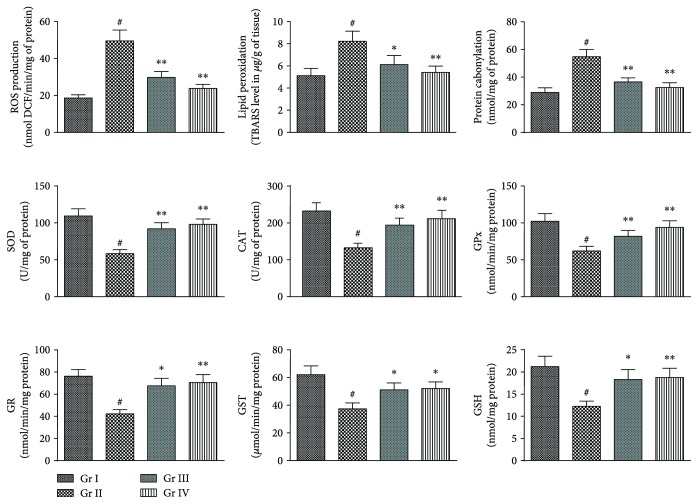
The effect on ROS accumulation, lipid peroxidation, protein carbonylation, and endogenous redox systems in the absence (NaAsO_2_) and presence of CA (NaAsO_2_ + CA) *in vivo* in the liver of experimental mice. Values are represented as mean ± SD (*n* = 6). ^#^Values significantly (*p* < 0.01) differ from normal control. ^#^Values significantly (*p* < 0.01) differ from normal control. ^∗^Values significantly (*p* < 0.05) differ from toxic control. ^∗∗^Values significantly (*p* < 0.01) differ from toxic control. SOD unit “U” is defined as inhibition (*μ* moles) of NBT-reduction/min. CAT unit “U” is defined as H_2_O_2_ consumption/min. Gr I: normal control; Gr II: toxic control; Gr III: CA (10 mg/kg) + NaAsO_2_ (10 mg/kg), Gr IV: CA (20 mg/kg) + NaAsO_2_ (10 mg/kg).

In *in vivo* assay, NaAsO_2_ (10 mg/kg) treated mice exhibited significant (*p* < 0.01) elevation in the level of intracellular ROS within the hepatic tissue (Figure 7). However, simultaneous administration of CA (10 and 20 mg/kg) along with NaAsO_2_ (10 mg/kg) could significantly (*p* < 0.01) attenuate intracellular ROS production in the liver (Figure 6). Significant (*p* < 0.01) upregulations in lipid peroxidation and protein carbonylation were observed in the liver of NaAsO_2_ (10 mg/kg) exposed mice. On the other hand, CA (10 and 20 mg/kg) treatment could significantly (*p* < 0.05–0.01) reinstate lipid peroxidation and protein carbonylation in the hepatic tissue of the experimental mice to near-normal status (Figure 7). NaAsO_2_ (10 mg/kg) further potentiated oxidative stress via significant (*p* < 0.01) depletion in the levels of GSH and endogenous antioxidant enzymes (SOD, CAT, GPx, GR, and GST) in the hepatic tissue (Figure 7). However, simultaneous administration of CA (10 and 20 mg/kg) along with NaAsO_2_ (10 mg/kg) could significantly (*p* < 0.05–0.01) revert GSH and endogenous antioxidant enzymes to near-normal status (Figure 7).

#### 3.2.4. Effects in the Expressions of Signal Proteins in the Liver

**Figure 8:**
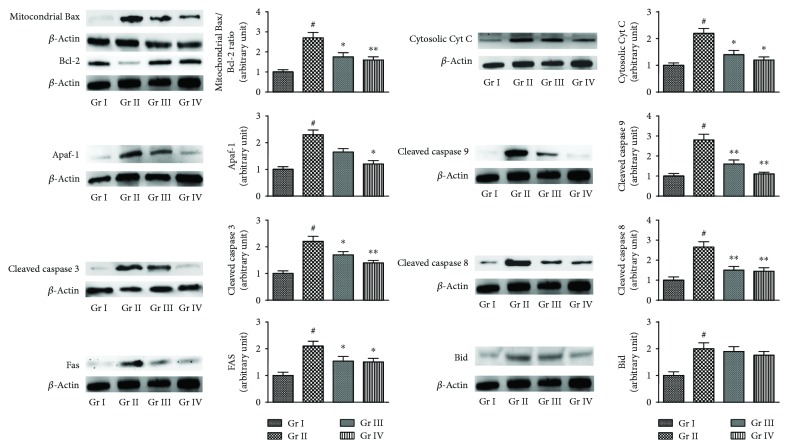
The effect on intrinsic and extrinsic apoptotic signaling in the absence (NaAsO_2_) and presence of CA (NaAsO_2_ + CA) *in vivo* in the liver of experimental mice. The relative band intensities were assessed and the normal control band was allotted a random value of 1. *β*-actin served as loading control. Values are expressed as mean ± SD (*n* = 6). ^#^Values significantly (*p* < 0.01) differ from normal control. ^∗^Values significantly (*p* < 0.05) differed from toxic control. ^∗∗^Values significantly (*p* < 0.01) differed from toxic control. Gr I: normal control; Gr II: toxic control; Gr III: CA (10 mg/kg) + NaAsO_2_ (10 mg/kg), Gr IV: CA (20 mg/kg) + NaAsO_2_ (10 mg/kg).

In this study, the expressions of different apoptotic proteins in the liver of experimental mice were assessed by Western blotting (Figure 8). Significant upregulations (*p* < 0.01) in the expressions of proapoptotic Bax protein in the mitochondria and concomitant downregulations (*p* < 0.01) in the expressions of antiapoptotic-Bcl-2 protein in the cellular fraction were observed in the liver of NaAsO_2_ (10 mg/kg) treated mice. In this study, significant (*p* < 0.01) high mitochondrial Bax/Bcl-2 ratio was observed in the hepatic tissue of NaAsO_2_ (10 mg/kg) treated mice. However, treatment with CA (10 and 20 mg/kg) along with NaAsO_2_ (10 mg/kg) could significantly (*p* < 0.05–0.01) reciprocate As-mediated alteration of Bax/Bcl-2 ratio in the liver of experimental animals. NaAsO_2_ (10 mg/kg) treated mice revealed significant (*p* < 0.01) upregulation of cytosolic cytochrome C in the hepatic tissue, which suggested the release of cytochrome C into cytosol from mitochondria. On other hand, treatment with CA (10 and 20 mg/kg) along with NaAsO_2_ (10 mg/kg) could significantly (*p* < 0.05) attenuate As-provoked cytochrome C release to cytosol. Significant upregulations (*p* < 0.01) in the expressions of Apaf-1 were recorded in the liver of NaAsO_2_-exposed mice. CA (20 mg/kg) treatment along with NaAsO_2_ (10 mg/kg) could significantly (*p* < 0.05) downregulate the expression of Apaf-1 in the hepatic tissue of experimental mice. NaAsO_2_ (10 mg/kg) treated mice exhibited significant (*p* < 0.01) upregulation in the expressions of cleaved caspases 3 and 9 in the liver of experimental mice. However, treatment with CA (10 and 20 mg/kg) along with NaAsO_2_ (10 mg/kg) could significantly reciprocate the expressions of cleaved caspases 3 (*p* < 0.05–0.01) and 9 (*p* < 0.01) in the liver of experimental mice. To study the effect of NaAsO_2_ on death receptor-mediated apoptosis, immunoblot analysis of FAS, Bid and cleaved caspase 8 was performed. NaAsO_2_ (10 mg/kg) treated mice exhibited significant (*p* < 0.01) upregulation in the expressions of the FAS, Bid, and cleaved caspase 8 in the hepatic tissue, which advocated simultaneous engrossment of the extrinsic pathway of apoptosis. Treatment with CA (10 and 20 mg/kg) along with NaAsO_2_ (10 mg/kg) could significantly reciprocate the As-mediated alteration in the expressions of FAS (*p* < 0.05) and cleaved caspase 8 (*p* < 0.01). However, CA (10 and 20 mg/kg) treatment did not exhibit any significant change in As-provoked Bid expression in the liver of experimental animals.

**Figure 9:**
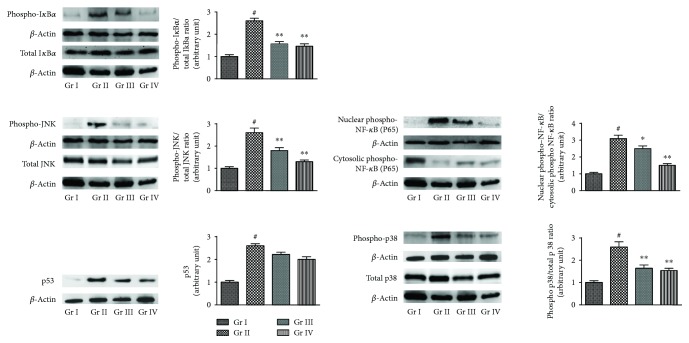
The effect on I*κ*Bα, NF-*κ*B, JNK, p38, and p53 signaling in the absence (NaAsO_2_) and presence of CA (NaAsO_2_ + CA) *in vivo* in the liver of experimental mice. The relative band intensities were assessed and the normal control band was allotted a random value of 1. *β*-actin served as loading control. Values are expressed as mean ± SD (*n* = 6). ^#^Values significantly (*p* < 0.01) differ from normal control. ^∗^Values significantly (*p* < 0.05) differed from toxic control. ^∗∗^Values significantly (*p* < 0.01) differed from toxic control. Gr I: normal control; Gr II: toxic control; Gr III: CA (10 mg/kg) + NaAsO_2_ (10 mg/kg), Gr IV: CA (20 mg/kg) + NaAsO_2_ (10 mg/kg).

To investigate the effect on NF-*κ*B, the signaling, NaAsO_2_ (10 mg/kg) treatment could significantly (*p* < 0.01) stimulate the phosphorylation of cytosolic I*κ*B*α* and thereby could significantly trigger the translocation of phosphorylated NF-*κ*B (p 65) (*p* < 0.01) to the nucleus (Figure 9). However, CA (10 and 20 mg/kg) along with NaAsO_2_ (10 mg/kg) could significantly (*p* < 0.05–0.01) attenuate the aforementioned NF-*κ*B signaling pathway in the liver of experimental mice (Figure 9). In this study, NaAsO_2_ (10 mg/kg) could significantly (*p* < 0.01) upregulate the phosphorylation of JNK and p38 (Figure 9). The treatment with CA (10 and 20 mg/kg) along with NaAsO_2_ (10 mg/kg) could significantly (*p* < 0.01) reciprocate the phosphorylation of JNK and p38 in the hepatic tissue of experimental mice (Figure 9). However, CA (10 and 20 mg/kg) could not counteract with the NaAsO_2_ (10 mg/kg) mediated p53 upregulation in the liver of experimental mice (Figure 9).

#### 3.2.5. Effects on ATP Levels, DNA Fragmentation, and DNA Oxidation in the Liver

**Figure 10:**
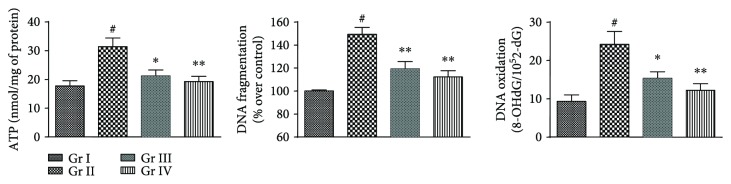
The effect on ATP, DNA fragmentation and DNA oxidation in the absence (NaAsO_2_) and presence of CA (NaAsO_2_ + CA) *in vivo* in the liver of experimental mice. Values are expressed as mean ± SD (*n* = 6). ^#^Values significantly (*p* < 0.01) differ from normal control. ^∗^Values significantly (*p* < 0.05) differ from toxic control. ^∗∗^Values significantly (*p* < 0.01) differ from toxic control. Gr I: normal control; Gr II: toxic control; Gr III: CA (10 mg/kg) + NaAsO_2_ (10 mg/kg), Gr IV: CA (20 mg/kg) + NaAsO_2_ (10 mg/kg).

In this study, NaAsO_2_ (10 mg/kg) treatment caused significant (*p* < 0.01) elevation in the level of cytosolic ATP in the hepatic tissue of experimental mice (Figure 10). The experimental observation could be substantiated with the establishment of apoptotic incidence within the liver. NaAsO_2_ (10 mg/kg) mediated significant (*p* < 0.01) upregulations of DNA fragmentation and oxidation further confirmed the establishment of As-mediated cytotoxic events within the hepatic tissue of mice (Figure 10). On the other hand, concomitant treatment with CA (10 and 20 mg/kg) along with NaAsO_2_ (10 mg/kg) could significantly (*p* < 0.05–0.01) reduce cytosolic ATP level, DNA fragmentation, and DNA oxidation in the hepatic tissue of experimental mice close to the normal status (Figure 10).

#### 3.2.6. Effects on the Histology of the Liver

**Figure 11:**
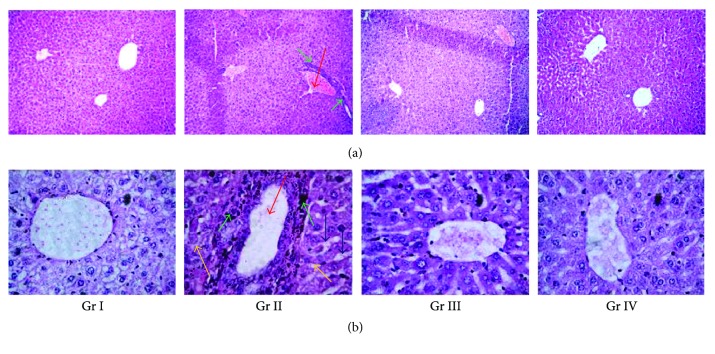
Histological sections 100x (a) and 400x (b) of the livers of experimental mice in the absence (NaAsO_2_) and presence of CA (NaAsO_2_ + CA). The liver sections of normal mice revealed normal portal vein and hepatocytes. NaAsO_2_-exposed liver section exhibited dilated portal vein (red arrow), vacuolated cytoplasm (blue arrows), apoptosis (yellow arrows), and leucocytes infiltration (green arrows) when compared with the section of normal control liver. CA treatment could reinstate NaAsO_2_ mediated the aforementioned pathological changed. Gr I: normal control; Gr II: toxic control; Gr III: CA (10 mg/kg) + NaAsO_2_ (10 mg/kg), Gr IV: CA (20 mg/kg) + NaAsO_2_ (10 mg/kg).

The histological sections of livers of experimental mice were depicted in Figures 11(a) and (b). The liver section of NaAsO_2_ (10 mg/kg) exposed mice exhibited diffused portal veins (red arrows), damaged hepatocytes with infiltrating leukocytes (green arrows), vacuolated cytoplasm (blue arrows), and induction of apoptosis (yellow arrows) when compared with the liver section of normal mice. However, CA (10 and 20 mg/kg) treatment could reinstate NaAsO_2_ (10 mg/kg) mediated the aforementioned pathological changes and restored the histology of liver to near-normal status.

### 3.3. In Silico Studies

#### 3.3.1. ADME Analysis

The drug-likeness and pharmacokinetic properties of CA was evaluated using QikProp module of Schrödinger. Drug-likeness and physicochemical properties were found to be as molecular weight = 332.4, H-bond donor = 3, H-bond acceptor = 3.5, predicted water partition coefficient = 3.7, aqueous solubility = −4.4, and total solvent accessible surface area = 575.8. The pharmacokinetic profiles, such as predicted brain/blood partition coefficient = −0.7, predicted apparent Caco-2 cell permeability = 182.7, number of likely metabolic reactions = 4, and human oral absorption = 89.3%, are significantly compliant with recommended values of drug-likeness. In Table 3, some other important predicted ADME parameters of CA were presented. The better drug-likeness with acceptable biopharmaceutical properties clearly indicated that CA may be a potential drug candidate.

#### 3.3.2. Molecular Docking

**Figure 12:**
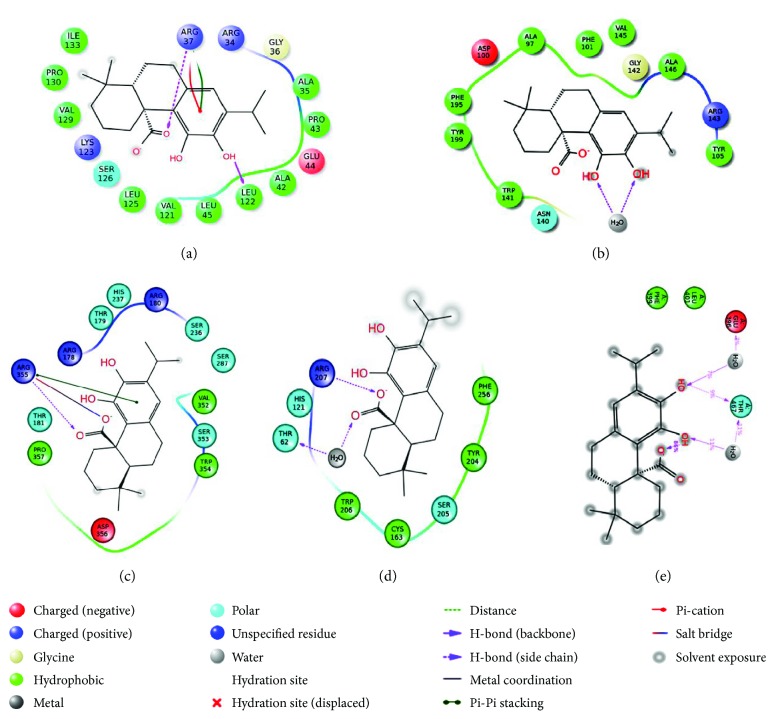
Docking interactions of CA with Bax (a), Bcl-2 (b), caspase 9 (c), caspase 3 (d), and caspase 8 (e) proteins.

Molecular docking analysis was attempted to dig into the possible binding patterns and interactions of CA with the selective signal proteins. Generated docking poses were ranked on the basis of Glide score. The lowest scored conformer was considered to be the best docking pose. Glide score represents the most capable best fit for a ligand in the active site of the target protein. In this study, Apaf-1 (PDB: 1Z6T), cytochrome C (PDB: 3ZCF), Bid (PDB: 4QVE), Fas (PDB: 3EZQ), and p53 (PDB: 2XWR) did not demonstrate significant docked pose after docking with CA; therefore, these proteins were discarded for further in silico analysis. Hydrogen bond (H-bond) interactions with the catalytic amino acid residues along with Glide score and Emodel values of other proteins were presented in Table 4. Molecular docking of Bax protein with the CA revealed three types of molecular interactions, namely H-bond, cation-*π*, and π-π stacking (Figure 12(a)). It was observed that H-bond interactions are fit into the Bax hydrophobic groove of Leu 122 residue and with the side chain of positively charged Arg 37 residue. The cation-π and π-π stacking interactions were also found with the same charged Arg 37 residue. In case of Bcl-2 protein, no single H-bond interaction was found to be formed with the catalytic residues. However, H-bond was predicted into ligand itself in the presence of H_2_O molecule through intramolecular H-bond interaction (Figure 12(b)). This intramolecular H-bond formation was also highlighted in the cocrystal structure of Bcl-2 (PDB: 4LXD). Docking analysis of caspase 9 revealed that Arg 355 residue participates to form H-bond with CA along with the formation of π-π stacking and salt bridge (Figure 12(c)). Since H-bond interaction with Arg 355 was also found with the ligand of X-ray crystal structure of caspase 9 (PDB: 2AR9), therefore, this interaction may be considered as a constraint to play a dynamic role in the biological phenomenon. The binding interactions of caspase 3 revealed that Thr 62 and Arg 207 residues are involved in H-bond formation with CA (Figure 12(d)). However, Thr 62 residue interacted through H_2_O-bridge formation. Caspase 8 was found to interact with two amino acid residues, Glu 396 and Thr 467, to form H-bond interaction with CA (Figure 12(e)). The same Glu 396 residue has been found to participate in H-bond formation in cocrystallized ligand of the PDB (4PRZ).

**Figure 13:**
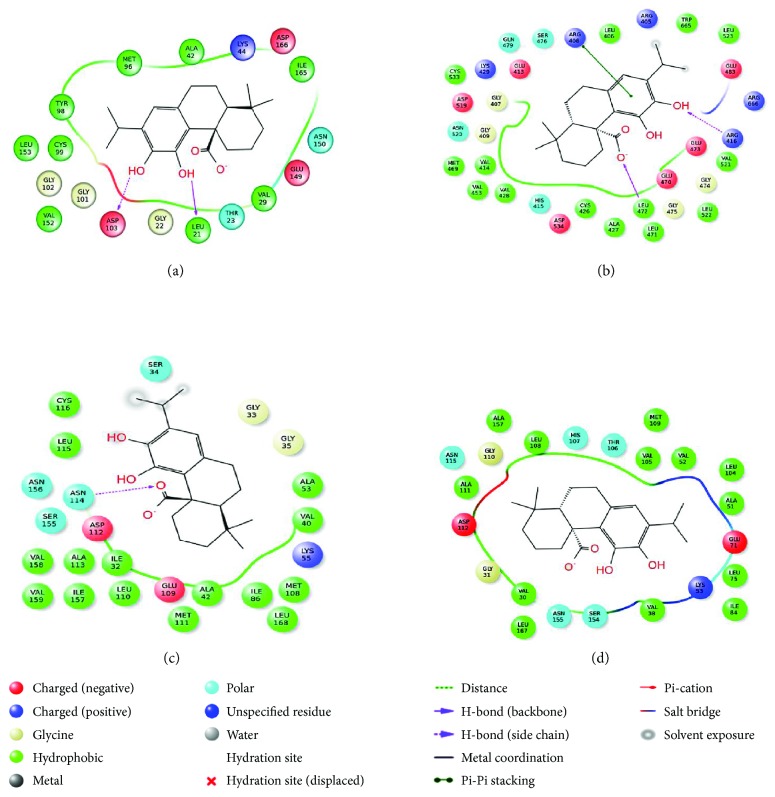
Docking interactions of CA with I*κ*B (a), NF-*κ*B (b), JNK (c), and p38 (d) proteins.

The binding interactions of CA with I*κ*B showed involvement of two residues, backbone atom of Leu 21 and side chain atom of Asp 103 in H-bond formation (Figure 13(a)). When comparing interaction with the cocrystallized structure of I*κ*B (PDB: 4KIK), it has been found that different amino acid residues (Glu 149, Glu 97, and Cys 99) were involved in H-bond formation. The docking analysis also revealed the predicted interaction for I*κ*B with negatively charged Asp 103 residue that might be important for the activation of the receptor. The H-bond interaction with two amino acids, Arg 416 (side chain) and Leu 472 (backbone), was observed between NF-*κ*B protein and CA along with π-π stacking interaction with Arg 408 residue (Figure 13(b)). The cocrystallized structure of NF-*κ*B (PDB: 4IDV) exhibited that Leu 472 residue is found to participate in H-bond interaction. Docking analysis of JNK exhibited a single H-bond interaction between Asn 114 and CA; however, the crystal structure of JNK (PDB: 4E73) specified the involvement of Met 111 in the interaction (Figure 13(c)). The interaction with Asn 114 might highlight that JNK has another binding site where CA interacted to form H-bond. For the protein p38, no H-bond interaction was observed but possessed moderate dock score of −5.608 kcal/mol in docking. Although, a number of lofty hydrophobic amino acids (Val 38, Ala 51, Val 52, Leu 75, Leu 104, Val 105, Leu 108, etc.) have been found to interact between CA and p38 (Figure 13(d)).

#### 3.3.3. Molecular Dynamics

**Figure 14:**
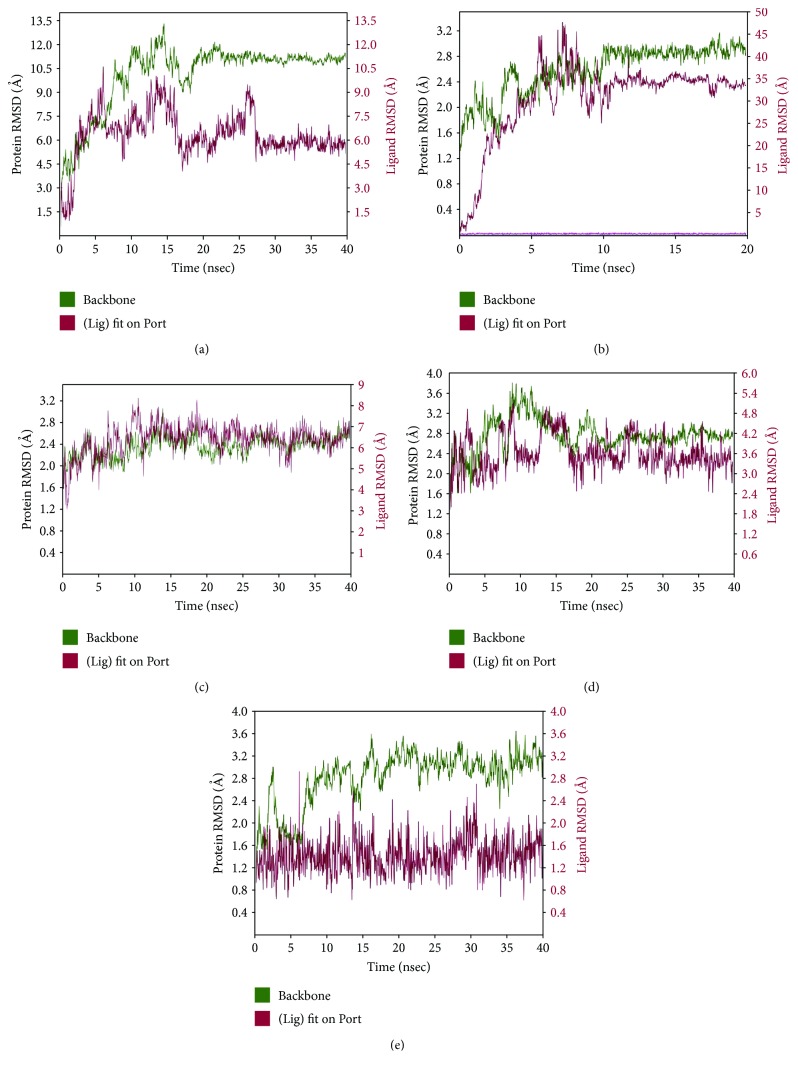
MD-simulated RMSD plot of Bax (a), Bcl-2 (b), caspase 9 (c), caspase 3 (d), and caspase 8 (e) backbones and CA ligand complex.

**Figure 15:**
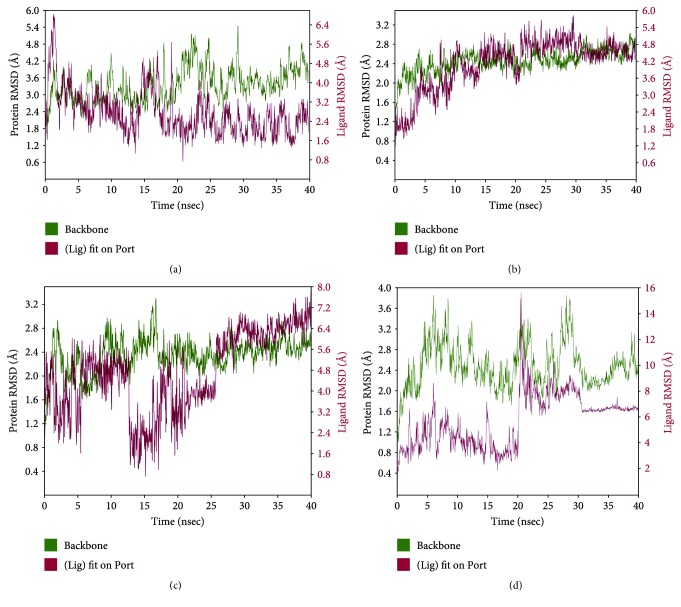
MD-simulated RMSD plot of I*κ*B (a), NF-*κ*B (b), JNK (c), and p38 (d) backbones and CA ligand complex.

The MD simulation was carried out for further refinement and stabilization of the docked complexes in dynamic environment to evaluate the most energetically stable binding conformation. The simulation time range of 20–40 ns was used to allow and permit reorganization of the interaction configuration of protein-ligand complex. In the simulation study, the compactness of each simulated complex has been analyzed through root-mean-square deviation (RMSD), root-mean-square fluctuation (RMSF), radius of gyration, and the secondary structure elements (SSE) of CA ligand interactions with the selective protein along with stable H-bond and hydrophobic interactions. The RMSD plot of Bax-CA complex showed that there is significant deviation till 30 ns of run. However, the complex was thereafter found to be appropriately stable and consistent until reaching to the end of the run (Figure 14(a)). The modeling analysis clearly appeared that the regions comprising catalytic amino acids of Bax protein interacting with CA exhibited adequate conformational stabilities. During the simulation run of Bcl-2-CA complex, the dynamic nature of interaction has been witnessed at 20 ns. The protein-ligand RMSD plot for Bcl-2-CA complex showed equilibrated simulation complex and stabilized at RMSD value of around 3 Å for protein backbone (Figure 14(b)). However, when the protein-ligand complex was first aligned on the protein backbone, the rearrangement of conformation explained a high deviation in RMSD of ligand with respect to time. Analysis of MD features of caspase 9-CA complex revealed the RMSD plot of MD complex with high fluctuation at the initial stage; however, after 32 ns, the complex become stabilizes gradually (Figure 14(c)). Analysis of the protein SSE, it has been noticed that a total of around 40.74% SSE contributed to the overall protein stability for caspase 9-CA complex. To realize the behavior of caspase 3-CA complex in dynamic environment, the MD simulation was carried out at 40 ns to study the molecular interaction pattern of the simulated complex. It has been observed in the RMSD plot (Figure 14(d)) that most stable time period for caspase 3-CA complex is 30–40 ns of the simulation run. For the production of binding orientation of caspase 8-CA complex in MD-simulated state, the simulation was executed at 40 ns. It has been noted that the complex is sufficiently stable in the long run (7–40 ns). It was exhibited by RMSDs of about 3.2 and 2.0 Å for protein backbone and ligand, respectively (Figure 14(e)). The MD simulation of I*κ*B-CA ligand bound complex was performed up to 40 ns. The RMSD plot (Figure 15(a)) showed minimal fluctuations during simulation run; however, a consistent interaction has been observed across the MD simulation trajectory after 25 ns. In protein SSE, *α*-helices (45.15%) and *β*-strands (7.41%) were monitored throughout the simulation. An interaction pattern at different time points of MD simulation for NF-*κ*B and CA complex has been recorded for 40 ns (Figure 15(b)). Although fluctuation in the RMSDs plot has been observed up to 30 ns of run, but the fluctuation was reduced during the run at 30–40 ns. It suggested that complex is undergoing a large conformational change and it leads to the equilibrated state during the simulation. The MD simulation run at 40 ns for the JNK-CA complex has not shown the consistent interaction across the MD simulation trajectory, as depicted in RMSD plot (Figure 15(c)). To evaluate the dynamic stability of p38-CA complex subjected to MD simulation for 40 ns, the RMSD plot showed higher congeneric fluctuation followed by small and large peaks in between (Figure 15(d)). The fluctuation-differentiate extended throughout the conformational arrangement as supported by docked complex which has not generated any bond interaction.

## 4. Discussion

Arsenites-contaminated drinking water has been regarded as a major hazard over 70 countries in the world [4]. Inorganic arsenicals have been shown to induce cytotoxic effect to the various organs through generation of excess of intracellular ROS coupled with downregulation of endogenous redox defense [4]. Therefore, employing an antioxidant would be a therapeutic strategy to counteract with As-mediated toxic exhibitions. CA, a naturally occurring phenolic diterpene, has been reported to possess significant ROS scavenging and antioxidant activities [14, 15]. It is one of the antioxidant food additives. Dried rosemary is used as a food component in various recipes. Dried rosemary (0.4–2.5 g/serving) corresponds to 0.05–0.4 mg/kg of CA [37]. Rosemary extract (0.01–2 g/kg) is also used as flavouring in processed foods. Rosemary extract containing variable amount of CA (5–20%) was classified as a food additive by the European Commission [37, 38]. Through various rosemary supplements up to 1-2 mg/kg of CA is consumed by human per day [37].

Present studies have been executed to evaluate the probable prophylactic role of CA against As intoxication in the liver of experimental mice employing established *in vitro* and *in vivo* preclinical assays. Despite the mouse has a higher metabolic turnover than human, the mouse has similar metabolite profile as human adults [39]. The absorption, distribution, and excretion of trivalent As follow similar patterns in both mouse and human. Trivalent As follow similar metabolic pattern like arsenite → monomethylarsonate → dimethylarsenite in both human and mouse [40]. Besides, As-mediated dysfunction in hepatic endothelial cells follows similar redox signaling in both mouse and human [41]. Considering the association in phenotypic anchors namely adsorption, arsenic burden in tissues, metabolism, excretion, and altered gene expression between the As-exposed human and mouse [41], mouse model has been employed in this study.

Haematological and serum biochemical status give primary indications of toxicological proceedings within the body and/or in the precise organs. In this study, NaAsO_2_ exposure significantly reduced erythrocyte count and thereby exhibited a significant reduction in haemoglobin level in mice. As-induced oxidative haematotoxicity has been reported in earlier literatures [6, 42]. As experiences biotransformation in the hepatic tissues through binding with thiol group of proteins and thereby interferes with the integrity of the membrane of hepatocytes following leakage of AST and ALT into the sera [6]. Therefore, AST and ALT are accepted to be the markers for hepatotoxicity [6]. In this study, significant raise in the AST and ALT levels was observed in the sera of NaAsO_2_-exposed mice. Similarly, the high levels of LDH and CK in the sera confirmed the loss of membrane integrity due to NaAsO_2_ intoxication. The high levels of aforementioned tissue-specific enzymes confirmed the induction of hepatotoxicity by NaAsO_2_. On the other hand, CA treatment could significantly reinstate the levels of aforementioned tissue-specific enzymes in the sera, which primarily gave an impression of hepatoprotective effect of the compound.

The earlier explanations revealed that NaAsO_2_ exposure significantly promoted intracellular ROS generation in both *in vitro* and *in vivo* systems [7, 15, 43]. ROS have multiple targets to initiate and propagate toxicological events namely oxidative destruction of cellular macromolecules, assuaging the endogenous redox defense and endorsing the apoptotic event via transformation in the normal transcriptions of different signaling proteins [26]. In this study, NaAsO_2_ exposure caused significant production and accumulation of intracellular ROS, which finally exerted significant extent of cytotoxic effect. The ROS in excess directly expose peroxidative injury to the membrane lipids, carbonylation of cellular proteins, and oxidation of nucleic acids [21]. However, CA could significantly attenuate the ROS-mediated toxicosis to the cellular biomolecules. The experimental observation would be correlated to the restriction of ROS production and/or ROS scavenging effect by CA.

Cellular redox defense systems play an imperative role to neutralize oxidative stress [44]. Cellular antioxidant enzymes and GSH constitute endogenous redox defense reservoir [44]. NaAsO_2_ exposure further endorse redox stress through significant inhibition of endogenous redox defense systems, which was in accordance to the previous observations [1, 6, 7]. Decrease in the levels of SOD and CAT could be accounted to an overproduction of superoxide anion during As metabolism [45]. Arsenites have high affinity toward thiol group [46], which would be corroborated to the depletion of cellular GSH levels. GSH acts as the substrate for the enzymes, specifically GR, GPx, and GST. The downregulation in the activities of GR, GPx, and GST would be correlated to the strong affinity of arsenites toward thiol group. Besides, GSH is also known to be a key intracellular reductant for As methylation and transport and thereby facilitate the removal of As from the body [47]. GSH-As complexes act as substrates for the ATP-binding cassette membrane transporters, which facilitate active efflux of As from the cells [48]. Depletion of hepatic GSH helps accumulation of As in the liver and promotes oxidative stress. On the other hand, CA along with NaAsO_2_ could significantly improve the cellular redox defense via augmenting the levels of endogenous antioxidant enzymes and GSH. CA treatment also promoted As clearance from hepatic tissue, which would be correlated with the CA-mediated upregulation of hepatic GSH level. The probable mechanism of CA has been proposed to be the conversion of catechol type-CA to quinone type-CA through donation of a pair of electrons to oxygen radicals to become electrophilic [49]. This quinone type-CA has been reported to form adducts with the -SH of GSH and/or other protein/enzymes and thereby activate antioxidant-responsive element. This antioxidant-responsive element further induces phase 2 enzymes and increases GSH level [49].

ROS has been reported to endorse apoptosis via alteration in the expressions of different signal proteins from their normal transcriptional levels [26]. ATP supplies the required energy for this programmed cell death [26]. In this study, a significantly high level of cytosolic ATP in the hepatic tissue followed by NaAsO_2_ exposure revealed the favorable cellular atmosphere for the cleavages of caspases in the cytosol. The augmentation of cytosolic ATP may be correlated to that of NaAsO_2_-mediated downregulation of microsomal ATPases in the liver [50]. Apoptosis is usually governed by the complex reciprocity between the pro- and antiapoptotic events via the transcription of respective signal proteins. Bax is a proapoptotic member of Bcl-2 family, which regulates apoptosis through mitochondrial stress. Translocation of Bax protein into mitochondria from cytosol initiates intrinsic apoptotic signaling [51]. In this study, NaAsO_2_ intoxication caused activation of proapoptotic signaling and mitigation, which was apparent from significant upregulation of Bax protein in mitochondria. Besides, NaAsO_2_ could significantly inhibit the expression of Bcl-2 in cytosol, which is a member of antiapoptotic protein [21]. The Bax translocation further opens the transition pores in the mitochondrial membranes resulting with the release of cytochrome C into the cytosol [26]. Cytochrome C release would be further potentiated through the downregulation of Bcl-2 in the cytosol [52]. In this study, a significant upregulation in the expression of cytochrome C in the cytosol was observed in NaAsO_2_-exposed hepatic cells. The release of cytochrome C into cytosol provides a key signal for the intrinsic pathway of apoptosis [52]. Through the interaction with Apaf-1, cytochrome C potentiates the downstream apoptotic signaling cascades via cleavages of caspases 9 and 3 into their respective cleaved/active forms in cytosol [52, 53]. It has been regarded that cytochrome C release is distal to caspase 8 activation in Fas-mediated apoptosis [52]. Earlier investigation revealed that, ROS can potentiate the death-receptor mediated apoptosis through receptor clustering and establishment of lipid raft-derived signaling platforms [54]. The Fas signaling is one of the key processes in extrinsic/death receptor-mediated apoptosis. In the extrinsic pathway of apoptosis, the activation of FAS resulted in the cleavage of procaspase 8 into its cleaved/active form followed by activation of Bid signaling [55]. In this study, significant upregulation in the expressions of FAS, cleaved caspase 8, and Bid were observed in NaAsO_2_-exposed hepatic cells. CA treatment could significantly attenuate the intrinsic and extrinsic pathways of apoptosis via reciprocating the expression of the involved transcription factors except Bid. NF-*κ*B is one of the redox-sensitive transcription proteins. ROS significantly contributes in signal-transduction pathways of NF-*κ*B [21]. The NF-*κ*B signaling is initiated with the phosphorylation of I*κ*B*α* on Ser 32 followed by translocation of phospho-NF-*κ*B (Ser 536) to the nucleus [56]. Nuclear translocation of phospho-NF-*κ*B can trigger proapoptotic event via promotion of Bax translocation to mitochondria. NF-*κ*B can also potentiate the expression of Fas-ligand and thereby exert a proapoptotic role [57]. In this study, NaAsO_2_ significantly upregulated NF-kB signaling and that could be attenuated by CA treatment. Among the many signaling pathways, mitogen-activated protein kinase (MAPK) family proteins are crucial for the maintenance of cells. C-Jun N-terminal kinase (JNK) and p38 are stress responsive signal proteins involved in apoptosis [58]. MAPK member proteins are also redox sensitive [21]. Strong functional interactions exist between the p53 and MAPK [59]. The activation of p53 can lead to cell cycle arrest [60]. Earlier observation revealed that, p38, JNK, and p53 significantly contribute in arsenite-induced apoptosis via activation of proapoptotic signaling in the mitochondria [60, 61]. In this study, significant upregulation in the p38, JNK, and p53 signaling was observed in NaAsO_2_-exposed hepatic cells. CA could significantly attenuate NaAsO_2_-mediated p38 and JNK signaling via preventing their phosphorylation. However, CA did not show significant control on p53 signaling in the hepatic cells of mice. Experimental observation revealed that CA exerted antiapoptotic effect through inhibition of MAPK activation, imbalance of pro- and antiapoptotic factors, and inhibition of caspase cleavage.

Utilizing chemometric analysis, valuable information on molecular basis has been elucidated for different protein-ligand complex structures in silico. In this study, Bax, Bcl-2, caspase 9, caspase 3, caspase 8, I*κ*B, NF-*κ*B, JNK, and p38 demonstrated significant interactions with CA in silico, which were in accordance to our observation in *in vitro* and *in vivo* studies. On the other hand, Apaf-1, cytochrome C, Bid, Fas, and p53 did not demonstrate significant docked pose. In our experiment, we did not observe any effect of CA on Bid and p53 expressions. However, CA has been found to regulate the expressions of Apaf-1, cytochrome C, and Fas *in vitro* and *in vivo*. The H-bond formation which is a key interaction to explicate the stability of the CA inside the binding pocket has been observed for most of the proteins, except Bcl-2 and p38. The *π*-π stacking, a noncovalent interaction, has been observed with Bax, caspase 9, and NF-*κ*B proteins. It might play vital roles in stabilization of protein structures and therefore important in many aspects of macromolecular study. The π-π stacking arrangement has been previously observed in many protein crystal structures as this arrangement is conserved in some families of enzymes. The occurrence of cation-π interaction, another type of noncovalent bonding, has been identified with positively charged residue in Bax protein. This interaction has been regarded as the most important interaction in structural biology. It plays a vital role in molecular recognition and signaling. MD simulations for all the complexes indicate that most interactions appeared in docking analysis are stable during MD simulation except JNK and p38. Thus, CA may be considered as a potential drug candidate having a wide range of bioactivity and may provide multitherapeutic benefits.

## 5. Conclusion

**Figure 16:**
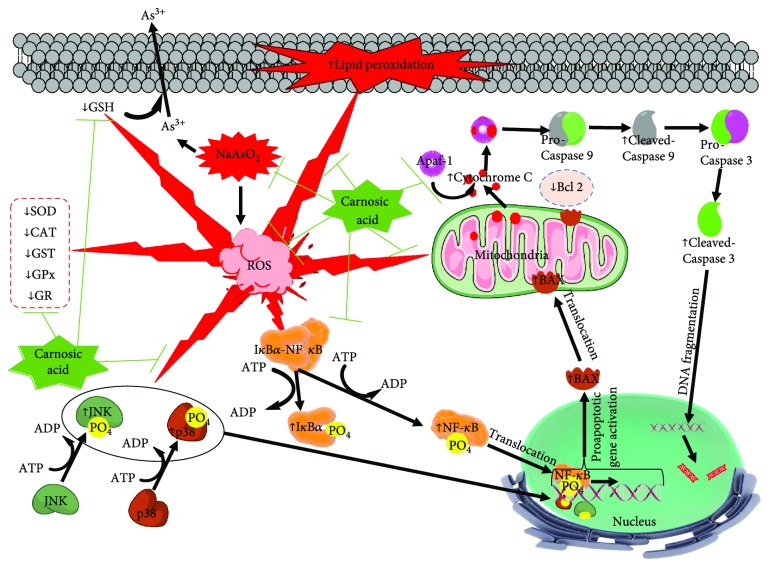
Schematic presentation probable protective mechanism of CA against NaAsO_2_-mediated hepatic injury. The red lightning bolts indicated the pathological events involved within NaAsO_2_-exposed hepatic cells. The green lines denoted the activity restricted by CA.

NaAsO_2_-mediated production and accumulation of oxidative free radicals play significant role in the As-induced hepatotoxicity. The present study demonstrated that NaAsO_2_ can elicit hepatocellular apoptosis by triggering NF-*κ*B, MAPK, p53, and intrinsic and extrinsic apoptotic signaling. On the other hand, treatment with the CA could significantly attenuate NaAsO_2_-mediated hepatotoxicity by counteracting with oxidative stress, promoting endogenous redox defense, accelerating As clearance and downstream regulation of apoptotic signaling cascades as observed in *in vitro* and *in vivo* preclinical assays (Figure 16). In silico molecular docking studies predicted the possible interactions between CA and the active sites of signal proteins. In silico ADME prediction revealed that CA supports the drug-likeness character ostensible from Lipinski's rule of five. Therefore, CA would have a good possibility to be a new therapeutic agent to counteract with As-mediated toxic manifestations.

## Figures and Tables

**Table 1:** Effects on body weight, liver weight, hepatic As content, and urinary As content in the absence (NaAsO_2_) and presence of CA (CA + NaAsO_2_) in mice.

Parameters	Gr I	Gr II	Gr III	Gr IV
Body weight (g)	28.16 ± 3.11	21.77 ± 2.08^#^	26.08 ± 3.01^∗^	26.92 ± 3.33^∗^
Liver weight (g)	1.24 ± 0.21	1.15 ± 7.29	1.17 ± 0.13	1.22 ± 0.25
Liver As (*μ*g/g of tissue)	0.06 ± .0001	0.51 ± 0.04^#^	0.35 ± 0.04^∗^	0.32 ± 0.02^∗^
Urinary As (*μ*g/g of creatinine)	2.34 ± 0.31	19.65 ± 2.17^#^	23.67 ± 2.98^∗^	26.01 ± 3.22^∗∗^

Values are expressed as mean ± SD (*n* = 6). ^#^Values differ significantly from normal control (*p* < 0.01). ^∗^Values differ significantly from NaAsO_2_ control (*p* < 0.05). ^∗∗^Values differ significantly from NaAsO_2_ control (*p* < 0.01). Gr I: normal control; Gr II: toxic control; Gr III: CA (10 mg/kg) + NaAsO_2_ (10 mg/kg); Gr IV: CA (20 mg/kg) + NaAsO_2_ (10 mg/kg).

**Table 2:** Effects on haematological and serum biochemical parameters in the absence (NaAsO_2_) and presence of CA (CA + NaAsO_2_) in mice.

Parameters	Gr I	Gr II	Gr III	Gr IV
Total erythrocyte count (×10^6^/mm^3^)	6.28 ± 0.42	3.22 ± 0.34^#^	4.04 ± 0.25^∗^	5.19 ± 0.67^∗∗^
Haemoglobin (g/dl)	8.76 ± 0.72	4.89 ± 0.43^#^	6.12 ± 0.48^∗^	6.88 ± 0.87^∗∗^
ALT (IU/I)	68.54 ± 5.81	112.98 ± 9.63^#^	89.85 ± 7.62^∗∗^	82.17 ± 6.15^∗∗^
AST (IU/I)	58.12 ± 4.63	82.91 ± 7.29^#^	73.62 ± 5.82^∗^	64.57 ± 5.51^∗∗^
CK (IU/mg protein)	10.87 ± 1.05	18.65 ± 1.24^#^	14.22 ± 0.98^∗∗^	13.78 ± 1.33^∗∗^
LDH (U/l)	171.34 ± 13.42	252.67 ± 21.50^#^	198.33 ± 20.12^∗∗^	187.54 ± 17.72^∗∗^

Values are expressed as mean ± SD (*n* = 6). ^#^Values differ significantly from normal control (*p* < 0.01). ^∗^Values differ significantly from NaAsO_2_ control (*p* < 0.05). ^∗∗^Values differ significantly from NaAsO_2_ control (*p* < 0.01). Gr I: normal control; Gr II: toxic control; Gr III: CA (10 mg/kg) + NaAsO_2_ (10 mg/kg); Gr IV: CA (20 mg/kg) + NaAsO_2_ (10 mg/kg).

**Table 3:** Prediction of drug-likeness and ADME profiles of CA.

Sl. number	Descriptors	Predicted values for CA	Recommended values (based on properties of 95% of known drugs)
1.	Molecular weight	332.4	130.0 to 725.0
2.	SASA	575.8	300.0 to 1000.0
3.	WPSA	0.0	0.0 to 175.0
4.	H-bond donor	3.0	0.0 to 6.0
5.	H-bond acceptor	3.5	2.0 to 20.0
6.	glob	0.8	0.75 to 0.95
7.	QPlogPo/w	3.7	−2.0 to 6.5
8.	QPlogS	−4.4	−6.5 to 0.5
9.	QPlogHERG	−1.9	Concern below −5
10.	QPPCaco	182.7	25 poor; >500 great
11.	QPlogBB	−0.7	−3.0 to 1.2
12.	QPPMDCK	100.2	<25 poor, >500 great
13.	QPlogKp	−3.2	−8.0 to −1.0
14.	#metab	4.0	1.0 to 8.0
15.	QPlogKhsa	0.3	−1.5 to 1.5
16.	% of human oral absorption	89.3	>80% is high; <25% is poor

SASA: total solvent accessible surface area; WPSA: weakly polar component of the SASA; glob: globularity descriptor; QPlogPo/w: predicted octanol/water partition coefficient; QPlogS: predicted aqueous solubility; QPlogHERG: predicted IC_50_ value for blockage of HERG K+ channels; QPPCaco: predicted apparent Caco-2 cell permeability; QPlogBB: predicted brain/blood partition coefficient; QPPMDCK: predicted apparent MDCK cell permeability in nm/sec; QPlogKp: predicted skin permeability; #metab: number of likely metabolic reactions; QPlogKhsa: prediction of binding to human serum albumin.

**Table 4:** Dock score, Emodel value, and interacting residues in molecular docking analysis.

Sl. number	Proteins	Glide scores (Kcal/mol)	Glide Emodel values	Interacting residues in H-bond interaction	Other interactions
1.	Bax	−3.115	−15.341	Arg 37, Leu 122	Cation-*π*, *π*-π stacking with Arg 37
2.	Bcl-2	−4.986	−29.586	—	H-bond with H_2_O
3.	Caspase 9	−3.596	−23.725	Arg 355	π-π stacking, salt bridge with Arg 355
4.	Caspase 3	−4.263	−30.352	Arg 207, Thr 62	—
5.	Caspase 8	−4.060	−19.905	Glu 396, Thr 467	—
6.	I*κ*B	−6.524	−33.382	Asp 103, Leu 21	—
7.	NF-*κ*B	−6.042	−36.121	Arg 416, Leu 472	π-π stacking with Arg 408
8.	JNK	−4.642	−17.079	Asn 114	—
9.	p38	−5.608	−30.359	—	—

## Supplementary Materials

Supplementary Materials Supplementary Figure 1: effect of NaAsO_2_ at different concentrations on cell viability in isolated murine hepatocytes. Values are expressed as mean ± SD (*n* = 3). Supplementary Figure 2: effect of CA to the histological structure of livers of experimental mice. Supplementary Table 1: the effect of CA (4 *μ*M) on ROS accumulation, lipid peroxidation, protein carbonylation, and endogenous redox systems in isolated murine hepatocytes. Supplementary Table 2: effects of CA (10 and 20 mg/kg) on haematological, serum biochemical, and redox parameters in mice.
