# Supplementary material for: Carnosic Acid, a Natural Diterpene, Attenuates Arsenic-Induced Hepatotoxicity via Reducing Oxidative Stress, MAPK Activation, and Apoptotic Cell Death Pathway
Source: Oxid Med Cell Longev. 2018 May 2;2018:1421438. doi: 10.1155/2018/1421438 (PMC5954942; doi:10.1155/2018/1421438)
Supplement: Supplementary Materials — Supplementary Figure 1: effect of NaAsO2 at different concentrations on cell viability in isolated murine hepatocytes. Values are expressed as mean ± SD (n = 3). Supplementary Figure 2: effect of CA to the histological structure of livers of experimental mice. Supplementary Table 1: the effect of CA (4 μM) on ROS accumulation, lipid peroxidation, protein carbonylation, and endogenous redox systems in isolated murine hepatocytes. Supplementary Table 2: effects of CA (10 and 20 mg/kg) on haematological, serum biochemical, and redox parameters in mice. [file 1421438.f1.pdf]

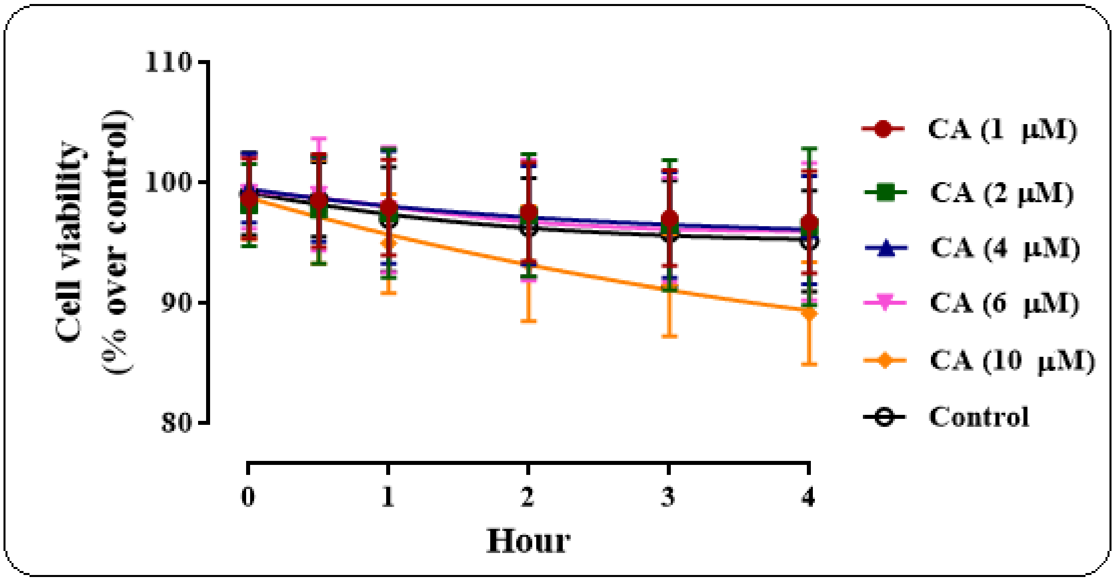

Supple. Figure 1. Effect of $NaAsO_2$ at different concentrations on cell viability in isolated murine hepatocytes. Values are expressed as mean ± SD (n = 3).

Supple Table 1. The effect of CA (4 µM) on ROS accumulation, lipid peroxidation, protein carbonylation and endogenous redox systems in isolated murine hepatocytes.

**Groups**	**Control**	**CA (4 µM)**
ROS production (nmol DCF/min/mg of protein)	6.02 ± 0.52	5.82 ± 0.45
TBARS (µg/g of tissue)	4.22 ± 0.31	4.07 ± 0.22
Protein carbonyl (nmol/mg of protein)	45.82 ± 4.32	42.50 ± 3.89
SOD (U/mg of protein)	85.87 ± 5.86	88.24 ± 6.67
CAT (U/mg of protein)	223.72 ± 14.33	226.98 ± 12.48
GPx (nmol/min/mg of protein)	71.26 ± 3.12	72.50 ± 4.17
GR (nmol/min/mg of protein)	76.67 ± 5.27	78.04 ± 5.73
GST (mmol/min/mg of protein)	75.85 ± 4.01	75.40 ± 4.45
GSH (nmol/mg of protein)	8.01 ± 0.84	8.41 ± 0.77

Values are expressed as mean ± SD (n = 3). SOD unit, “U” is defined as inhibition (µ moles) of NBT-reduction/min. CAT unit “U” is defined as H_2O_2 consumption/min.

Supple Table 2. Effects of CA (10 and 20 mg/kg) on haematological, serum biochemical and redox parameters in mice.

Parameters	Control	CA (10 mg/kg)	CA (20 mg/kg)
Total erythrocyte count (x $10^6/mm^3$)	6.45 ± 0.67	6.42 ± 0.51	6.64 ± 0.78
Haemoglobin (g/dl)	8.98 ± 0.92	8.75 ± 0.87	9.33 ± 0.98
ALT (IU/I)	64.48 ± 6.12	65.21 ± 5.19	64.77 ± 6.03
AST (IU/I)	58.33 ± 5.04	56.48 ± 6.13	57.11 ± 4.67
CK (IU/ mg protein)	10.24 ± 1.29	10.09 ± 0.96	9.89 ± 0.89
LDH (U/l)	167.43 ± 15.50	169.33 ± 14.29	161.81 ± 16.07
ROS production (nmol DCF/min/mg of protein)	15.84 ± 1.46	14.72 ± 1.37	14.18 ± 1.23
TBARS (µg/g of tissue)	5.32 ± 0.65	5.24 ± 0.43	5.19 ± 0.51
Protein carbonyl (nmol/mg of protein)	29.87 ± 2.44	27.99 ± 3.33	27.34 ± 2.68
SOD (U/mg of protein)	98.72 ± 8.11	101.87 ± 8.67	101.84 ± 9.94
CAT (U/mg of protein)	212.29 ± 22.54	215.67 ± 20.13	221.84 ± 18.75
GPx (nmol/min/mg of protein)	101.85 ± 8.19	103.08 ± 9.87	102.89 ± 8.71
GR (nmol/min/mg of protein)	76.18 ± 5.27	75.35 ± 5.46	77.23 ± 7.09
GST (mmol/min/mg of protein)	69.80 ± 5.21	70.52 ± 6.67	70.77 ± 6.55
GSH (nmol/mg of protein)	16.01 ± 1.37	16.17 ± 1.07	16.92 ± 1.49

Values are expressed as mean ± SD (n = 6). SOD unit, “U” is defined as inhibition (µ moles) of NBT-reduction/min. CAT unit “U” is defined as H_2O_2 consumption/min.

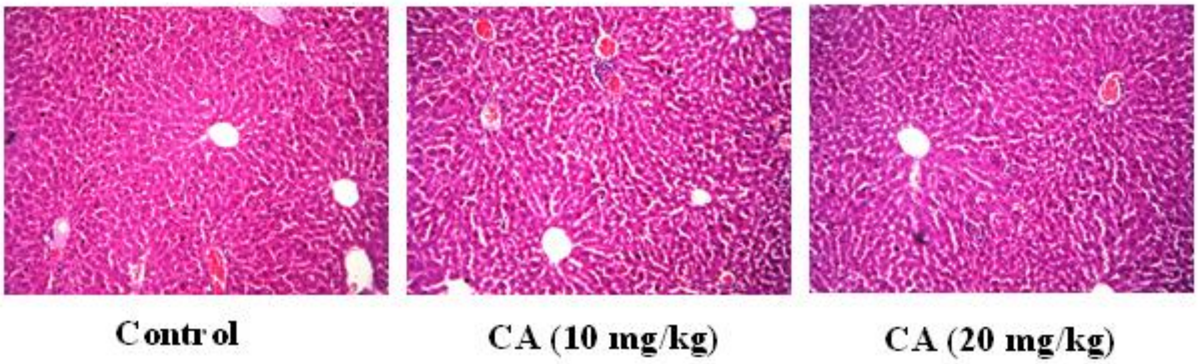

Supple. Figure 2. Effect of CA to the histological structure of livers of experimental mice.
